# What radiologists need to know about patients’ expectations: P.A.T.I.E.N.T.S C.A.R.E.R.S A.I.M.S

**DOI:** 10.1186/s13244-022-01184-w

**Published:** 2022-03-22

**Authors:** European Society of Radiology (ESR), Dominique Carrié, Cheryl Cruwys, Adrian Brady, Birgit Bauer, Andrew England, Nikoleta Traykova, Caroline Justich, Erik Briers, Judy Birch, Núria Bargalló Alabart, Andrea Rockall, Apostolos Karantanas, Carlo Catalano

**Affiliations:** Am Gestade 1, Vienna, Austria

**Keywords:** Radiology, Patient/doctor communication, Patient-centred radiology, Radiologists, Radiographers

## Abstract

The Patient Advisory Group (PAG) of the European Society of Radiology aims to highlight, in this short paper, patients’ expectations from the radiological community and support workers, throughout the patient’s medical imaging journey for completion of diagnostic or interventional examinations. In order to maintain constant awareness of patients’ expectations, key expectations have been summarised in an easy-to-remember mnemonic: **PATIENTS CARERS AIMS.**

Due to disparate healthcare systems and medical imaging services in Europe, not all patient expectations can be systematically met, but healthcare providers should be mindful, when setting up new operational procedures, of the need to focus on patient-centred needs and care. At times when new or improved technology is being introduced, such as artificial intelligence applications, telemedicine, robotisation of interventional procedures and digitised records, the impact on radiologist–patient communication and interactions should be considered.

**Supplementary Information:**

The online version contains supplementary material available at 10.1186/s13244-022-01184-w.

## Key points

- Radiology departments and medical imaging services are not always dedicated to patients’ needs and expectations.
- Patient-centred radiology is key to satisfactorily meeting patients’ demands.
- This ESR-PAG publication attempts to summarise the most important patients’ expectations and needs.
- The ‘PATIENTS CARERS AIMS’ statement can be used as a reminder to radiology department staff to work to provide patient-focussed services on a daily basis.

## Introduction

The European Society of Radiology Patient Advisory Group (ESR-PAG) was created in 2013 [1] and brings together different European representatives from patient groups, as well as radiologist and radiographer members of the various ESR committees. Its primary goals are to develop and maintain the relationship between radiologists–radiographers and patients, to improve patients’ knowledge about different imaging modalities and to advocate for a patient-centred approach to the practice of medical radiology in Europe. Since 2013, the ESR-PAG has been involved in numerous ESR initiatives, including the International Day of Radiology (IDoR), the ESR Patient Information website, the EuroSafe Imaging Campaign, contributions to ESR publications, the ESR-PAG social media team and the Esperanto Patient Satisfaction Questionnaire Audit [2]. Every year, at the European Congress of Radiology in Vienna, the ESR-PAG organises dedicated sessions, which provide a platform for listening to patient representatives and for dialogue with radiologists and radiographers.

Modern medical care relies heavily on diagnostic imaging studies and interventional procedures. In addition, the follow-up of multiple common chronic pathologies (e.g. cancer, neurologic and cardiovascular pathologies) requires patients to regularly visit imaging departments for further checks and consultations. The ESR-PAG works to develop patient-focused initiatives, and this paper aims to highlight, to the radiological community, the patients’ needs, in the form of a simple expression of principles underpinning patient expectations during their visit to an outpatient or inpatient medical imaging service.

The expectations that are outlined emerge along the patient's pathway in medical imaging, before, during and after the imaging examination. The principles outlined may form the basis for standards, which could be developed or incorporated into existing radiological standards and become the basis for future clinical audits.

## Before imaging examination: P.A.T.I.E.N.T.S

*Purpose* Patients should receive an explanation as to why the imaging is necessary.

*Advance* Providing the patient with information in advance gives them time to understand the usefulness, the benefits and potential harms of the recommended examination, as well as possible therapeutic consequences, especially in the case of interventional imaging. The patient is then better prepared and can make an informed decision when asked to give consent. Advance provision of information allows patients to consider and formulate questions they may wish to have answered when they visit the radiology facility for their investigation or procedure.

*Transparency* The known limitations of examinations should be explained to the patient (regardless of availability of modalities that can be offered). As Leonard Berlin (the noted US radiologist commentator on quality and medicolegal issues) observed, ‘radiologists can do an injustice to the patient by withholding our superior knowledge’ [3]. Some examples of information which should be made clear to patients include the limitation of mammography in patients with dense breasts, the limitations of a CT scan to screen for brain demyelinating lesions, the limitations of pelvic ultrasound in the diagnosis of endometriosis and possible associated risks such as consequences of radiation dose, radiation protection, magnetic protection, allergic reactions or impact on kidney function from intravenous contrast injections.

*Information* Ideally, the referring physician should provide explicitly clear, educational patient information prior to the examination. Patients’ value face-to-face communication with radiologists, which makes it easier to address their concerns about safety, quality of care and imaging procedures. Alternatively, when organising the appointment, the imaging centre/hospital secretarial/administration staff should communicate pre-appointment information either by telephone, in writing or by email, including links to imaging service websites, patient portals, video communication, etc. Digital health will give in the future news tools to improve communication between medical teams and patients.

*Equality* While the referring physician may identify and prioritise the urgency of imaging, patients’ views should be taken into account. Every patient matters and every patient is different. While patient care considerations include the severity of their condition, their level of pain, mobility, learning difficulties, etc., patients may, of course, be treated differently according to their needs, but one patient should not be treated more favourably than another. In other words, timely and equal access to imaging should be available to all without discrimination and should not be related to a person’s age, status, profession or other characteristics which do not derive from their medical need.

*Needs* The needs of patients must be considered [4]. For example, a patient who needs to contact their specialist (oncologist, neurologist, etc.) to discuss their status after having imaging performed, should be able to access the relevant administrative/professional staff to schedule an appointment, rather than passively awaiting communication initiated by their doctor.

*Trust* If effective pre-examination administrative procedures are in place prior to the appointment, the patient may feel a sense of trust in the run-up to the examination and may feel more comfortable knowing what to expect. Therefore, in order to harness patients’ trust [5], communication and exchange of information between the radiology department and patients is of the utmost importance, starting with the preparation for appointments [6]. Ultimately, such trust is a prerequisite to obtain the patient’s informed consent [7].

*Shared Decision-making* If patients are in possession of high quality, comprehensive health information (in appropriate, understandable language) [8, 9] they are better equipped to have informed conversations and to make shared decisions about their health [10].

## During the course of the imaging: C.A.R.E.R.S

*Comfort* The comfort of the facilities is important for patients, especially in waiting areas. Welcoming and calming premises may help to reduce pre-imaging stress sometimes experienced by patients [11]. Provision of adequate toilets, changing facilities and privacy are central to ensuring patient dignity is preserved.

*Attentiveness* The availability of staff to attend to patient's needs and provide support must be constant throughout the process. The sense of security felt by patients stems from prior explanations that have been provided, the patient’s consent to the imaging procedure, their confidence in the equipment being used, the presence and attentiveness of the staff, including the radiographers undertaking the examination for the patient, and where relevant, the supervising radiologist.

*Reassurance* Empathy, listening, patience and understanding are qualities that must be developed in all medical imaging teams, starting from the first person in contact with the patient, throughout the whole course of the examination, including waiting for results (if available). In many jurisdictions, the radiographer often interacts with the patient during their visit to the imaging department; the radiographer must provide a sense of safety, reassurance and empathy. It is imperative that radiologists recognise the importance of the radiographers’ role. The patient must feel that they are the focus of attention of care teams [12].

*Explanations* should be given throughout the examination, e.g. specific preparations for an examination, need for injection, radiographers’ expectations of the patient (e.g. mobility issues or frailty), updates on prolonged waiting times, the need for any further injection or any additional imaging with another modality, etc. By virtue of being close to the patient during the examination, radiographers have an essential role to play in providing these explanations.

*Results* The methods of delivery and timing of availability of results should be known by patients when booking the appointment. If results will not be immediately available, ideally the patient should be given either verbal or written notification of when they will receive the results. If result availability will be delayed, the reasons should be explained (for example, additional specialised advice required). Whenever possible, results should be available within a reasonable time frame [13]. The radiologist is an appropriate person to discuss the patient’s medical imaging outcomes, imaging limitations (if any) and if required, the need for further investigation and additional specialised advice. Some patients’ results will be transmitted to them by the referring hospital physician, others by their general practitioner and increasingly through secure teleconsultation. Many patients feel that the radiologist who supervised their examination should communicate these results directly to them [14, 15]. Ideally, radiologists should endeavour to make themselves available to discuss results at the patient’s request; where possible, this should be organised by the radiologist, conveniently, directly after the examination [16, 17]. Alternative radiologist–patient consultations can be by telephone or by teleconsultation. Specific consultations for the delivery of results have been proposed [18]. Such radiologist–patient consultations should ideally be coordinated with the referrer, to ensure consistent information provision to patients.

In an interventional context, patients must, of course, be informed of any problems that may occur as a result of the procedure, monitoring instructions, possible treatments and prescriptions to follow. Follow-up information sheets/leaflets should be given to the patient when they leave.

In some healthcare systems, unfortunately, patient–radiologist communication is either limited or does not exist, which may result in radiologists seeming ‘invisible’ [19, 20]. This is generally not related to unwillingness to engage, but instead often due to high volume of patients and/or workforce issues that can result in insufficient time being available to see patients [21].

The lack of contact with radiologists may have a detrimental effect on patient–radiologist communication, as many patients may be keen to have face-to-face discussions. Direct communication between radiologists and patients is also beneficial to radiologists, enhancing their appreciation of patients’ specific presentations and concerns.

Where possible, medical imaging reports should use lay/patient-friendly terminology and simplified patient reports should be provided in plain language more suited to a patient’s understanding, as opposed to scientific vocabulary that is understood only by medical professionals [8, 9].

*Safety* Patients should feel confident that they will be safe when attending a medical imaging examination or image-guided procedure, confident that they can access high-quality health care, avoiding adverse effects and inaccurate or delayed diagnoses. The COVID-19 pandemic has further emphasised that it is critical that radiology departments follow official guidance to keep patients safe.

## After the imaging examination: A.I.M.S

*Ascertain If Medical* Imaging was *Satisfactory:* Radiologists need to increase everyday patient involvement in the process of medical imaging and welcome patient group collaborations that can contribute to patients’ experience. Following imaging examinations, patients need to have the opportunity to express their level of satisfaction or dissatisfaction and to provide feedback via a patient satisfaction questionnaire. The Esperanto 2019 ESR Guide to Clinical Audit in Radiology and Clinical Audit Tool includes an example of a Patient Satisfaction questionnaire to improve imaging services and care in European countries [2].

Patient feedback should allow radiologists to better understand their patients' expectations.

## Conclusion

Radiology departments should try to review their existing and future organisation with the imaging team, incorporating the mnemonic, *PATIENTS CARERS AIMS (summarised in Additional file*: 1 poster) and work to apply these key points to benefit the patient—the most important stakeholder in radiology—with the aim to improve patient–radiologist communication in medical imaging in Europe, to ultimately meet patients’ expectations.

## Supplementary Information

**Additional file 1:** PATIENTS CARERS AIMS poster.

## Data Availability

All information is included in this statement.
