# Supplementary material for: What radiologists need to know about patients’ expectations: P.A.T.I.E.N.T.S C.A.R.E.R.S A.I.M.S
Source: Insights Imaging. 2022 Mar 22;13:53. doi: 10.1186/s13244-022-01184-w (PMC8938634; doi:10.1186/s13244-022-01184-w)
Supplement: Supplementary file 1 — Additional file 1: PATIENTS CARERS AIMS poster. [file 13244_2022_1184_MOESM1_ESM.pdf]

Always consider the needs of your *patients*

Purpose
- Do you inform your patients of the reason for the need for an imaging examination?

Advance
- Do you provide your patients with information about examinations in advance?

Transparency
- Do you inform your patients of the benefits of imaging & any potential harms and limitations?

Information
- Do you give clear and understandable patient information about examinations? Do you obtain patient consent?

Equality
- Do you provide fair and equal care?

Needs
- Do you think all the patients’ needs are met?

Trust
- Do you think your patients have trust in your staff and imaging equipment?

Shared Decision-making
- Do you involve your patients in their medical decisions?

Comfort
- Do you offer comfortable conditions and surroundings during the patients' visits?

Attentiveness
- Are your staff available to listen to all patients’ concerns?

Reassurance
- Do your staff try to calm patients’ fears and put them at ease?

Explanations
- Do your staff provide explanations during the imaging examination?

Results
- Do you give information about how and when patients can expect results?

Safety
- Do you think your patients feel safe?

AIMS

Ascertain If Medical imaging was Satisfactory

- Do you provide a patient questionnaire?
- Do you act upon responses received, in an effort to improve the services you provide?

ESR | PAG
EUROPEAN SOCIETY OF RADIOLOGY
